# Metabolic consequences and nailfold capillary changes in children with familial Mediterranean fever

**DOI:** 10.1186/s13052-025-01861-8

**Published:** 2025-02-07

**Authors:** Ahmed S. Abo Hola, Rania S. El Zayat, Wafaa Ahmed Shehata, Mai I. Elashmawy, Noha E. Khalaf, Heba M. S. El Zefzaf

**Affiliations:** 1 Department of Pediatrics, Faculty of Medicine, Menoufia University, Yassin Abdel-Ghafar Street, Shebin El-Kom, Shebin El-Kom, 32511 Menoufia Egypt; 2 Department of Dermatology, Andrology & STDs, Faculty of Medicine, Menoufia University, Shebin El-Kom, Egypt; 3 Clinical Pathology Department, National Liver Institute, Menoufia University, Shebin El-Kom, Egypt; 4 Egyptian Ministry of Health, Cairo, Egypt

**Keywords:** Atherogenic indices, Familial Mediterranean fever, Insulin resistance, Nailfold capillary changes, Serum lipid profile

## Abstract

**Background:**

There’s an increasing role of nailfold capillaroscopy in the evaluation of peripheral vascular disease in chronic inflammatory disorders. Familial Mediterranean fever (FMF) is one such disorder, which raises concerns about increased cardiovascular risk, with scarce data available in children. Therefore, we aimed to evaluate insulin resistance, lipid profile, atherogenic indices, and nailfold capillary (NC) changes in children with FMF.

**Methods:**

Fifty-four children diagnosed with FMF were evaluated by measuring complete blood count, ESR, CRP, serum amyloid A (SAA), Homeostatic Model Assessment for Insulin Resistance (HOMA-IR), lipid profile, and atherogenic indices, along with a nailfold capillaroscopic examination, both during acute attacks and attack-free periods.

**Results:**

During attack-free periods, patients exhibited higher total leucocytic counts, ESR, CRP, SAA, HOMA-IR, total cholesterol (TC), non-high-density lipoprotein cholesterol (non-HDL-C), Castelli’s risk index I (CRI I), and atherogenic coefficient (AC), and a lower hemoglobin level than controls. Additionally, the NC examination identified avascular areas in 14.8% of patients, tortuosities in 18.5%, enlargements in 14.8%, and microhemorrhages in 7.4%. These parameters showed significant increases during acute attacks. HOMA-IR showed positive correlations with TC, non-HDL-C, CRI I, and AC; however, NC changes were strongly connected with disease duration and SAA.

**Conclusions:**

Insulin resistance, alterations in serum lipids and atherogenic indices, and NC changes significantly endure in children with FMF during attack-free periods compared to controls, with more prominence during acute attacks. These parameters are linked to subclinical vascular injury and elevated cardiovascular risk, so their monitoring is crucial in these patients for early detection and intervention.

## Background

Familial Mediterranean fever (FMF) is an autosomal recessive autoinflammatory disease characterized by recurrent fever and serositis. Various studies have highlighted that subclinical inflammation in FMF patients may persist during attack-free periods, which may contribute to vascular endothelial dysfunction, considerably higher carotid intima-media thickness, and increased atherosclerotic risk [1–4].

Insulin resistance and dyslipidemia are two factors that contribute to the accelerated atherosclerosis seen in inflammatory rheumatic disorders such as rheumatoid arthritis and systemic lupus erythematosus [5]. Nevertheless, contradictory data are available in FMF.

Insulin resistance may contribute to subclinical vascular injury involving functional and structural damage to the arterial wall, with impaired vasodilation, reduced distensibility or even vascular calcification, leading to an increased cardiovascular risk [6]. In this instance, it has been reported that overt or subclinical inflammation in FMF may cause insulin resistance [7]; however, limited pediatric research has been carried out.

Moreover, epidemiological studies have shown that dyslipidemia alone is sufficient to drive the atherosclerotic process, even in the absence of other risk factors, and patients with FMF have been documented to have aberrant lipid profiles [8]. Furthermore, in recent years, atherogenic indices have emerged as indicators for atherosclerosis and cardiovascular disease [4, 9]. Therefore, monitoring these parameters is essential for early intervention regarding any potential cardiovascular risk.

Inflammatory disorders with vascular injury might cause abnormal nailfold capillary (NC) patterns, which may reflect the intensity and prognosis of underlying disease [10]. Capillaroscopy is an easy-to-use, low-cost, non-invasive method for assessing microvasculature, and in clinical practice, it is utilized as a helpful diagnostic tool for Raynaud’s phenomenon and scleroderma. Additionally, its role in vasculitis has been researched in systemic lupus erythematosus and inflammatory myopathies [11]. In FMF, vasculitis has been reported to occur [12]. However, little is known about NC changes in FMF patients, especially in pediatrics.

Since these parameters are associated with subclinical vascular injury and increased cardiovascular risk, our goal was to assess the potential of developing insulin resistance, alterations in the serum lipid profile and atherogenic indices, and NC changes in children with FMF. This would enable the early identification of patients with microangiopathy who require closer monitoring and more extensive therapy.

## Methods

### Patients

Following the Menoufia Faculty of Medicine’s Institutional Review Board approved the study (ID number: 12/2022PEDI19), 54 children diagnosed with FMF based on Eurofever/PRINTO clinical plus genetic criteria [13] were recruited from the Pediatric Immunology and Rheumatology Clinics of Menoufia University Hospitals between March 2023 and February 2024, along with 54 age- and sex-matched healthy children as controls. The following conditions excluded patients from the study: chronic autoinflammatory illness other than FMF, Raynaud’s phenomenon, vasculitis, malignancies, hypothyroidism, hyperlipidemia, obesity, hepatic or renal impairment, cardiovascular disease, and the use of medications other than colchicine.

The patients’ caregivers approved the consent letter, and all patients underwent a comprehensive clinical history emphasizing the age of onset of the disease, disease duration, symptoms (fever, abdominal pain, and joint pains), periodicity of disease acute attacks, treatment protocol, and results of Mediterranean fever (MEFV) gene mutation analysis. A thorough clinical examination was also carried out.

### Laboratory evaluation

Under aseptic conditions, venous blood samples were withdrawn in the morning on an empty stomach: once for controls and twice for patients during an acute attack and an attack-free period (defined as being symptom-free for at least 3 weeks). The Sysmex XT1800i Automated Hematology Analyzer was used to evaluate the complete blood count. C reactive protein (CRP), serum amyloid A (SAA), triglycerides (TG), total cholesterol (TC), low-density lipoprotein cholesterol (LDL-C), high-density lipoprotein cholesterol (HDL-C), fasting plasma glucose and fasting insulin levels were assayed from the serum using the Cobas 6000 analyzer, Roche Diagnostics, Switzerland.

Consequently, lipid profile data were used to acquire atherogenic indices, including atherogenic index of plasma “AIP” (Log TG/HDL-C), atherogenic coefficient “AC” (non-HDL-C/HDL-C), Castelli’s risk index I “CRI I” (TC/HDL-C), Castelli’s risk index II “CRI II” (LDL-C/HDL-C), and non-high-density lipoprotein cholesterol “non-HDL-C” (TC-HDL-C) [4, 14].

The Homeostatic Model Assessment for Insulin Resistance (HOMA-IR) was calculated from the measurements of fasting plasma glucose and fasting insulin levels; the following formula was applied: [fasting glucose (mmol/L) × fasting insulin (mIU/L)]/22.5 [15].

### Nailfold capillaroscopic examination

Using a standard dermatoscope (Dermalite DL4), a dermatologist with expertise in rheumatic diseases NC alterations performed nailfold capillaroscopy examinations on patients (during and after an attack) and controls. For fifteen minutes, each participant rested in the test room which had a room temperature between 20 °C and 22 °C. All 10 fingers of the hands were examined except for those with recent local trauma, with a transparent gel placed between the probe and the examined nailfold area to enhance image resolution. Subsequently, the pictures underwent registration and processing to determine whether avascular regions, microhemorrhages, enlargements, or tortuosities of the nailfold capillaries were present.

For scoring NC changes, avascular areas were scored as 0 for negativity and 2 for positivity. Tortuosities were scored as 0 if they represented less than 10% of capillary loops, 1 for more than 10%, and 2 for crossed loops or bushy capillaries. Capillary enlargements were scored as 0 for absence, 1 for focal or apical enlargements, and 2 if they were frequent or megacapillaries. Microhemorrhages were scored as 0 for absence, 1 for focal dotting, and 2 for frequent dotting or clustering [16].

### Statistical analysis

Qualitative data were presented in numbers and percentages. The Shapiro-Wilk test was used to verify the normality of the variables’ distribution. Mean ± standard deviation represented the normally distributed quantitative data, while the median (interquartile range) represented the non-normally distributed quantitative data. For comparing normally distributed data, the t-test was used, while the Mann-Whitney U test was used for non-normally distributed data. The Chi-square test analyzed the associations of qualitative variables. To conduct a paired difference test of repeated measurements, the Wilcoxon signed ranks test was used. The McNemar test checked the marginal homogeneity of two contrasted variables. Spearman and Pearson coefficients analyzed data correlations. A *p*-value ≤ 0.05 was considered statistically significant. All the analyses were performed using IBM SPSS Statistics for Windows, Version 20.0. (IBM Corp, Armonk, New York).

Statistics and the Sample Size Pro tool version 6 indicated that sample size to be at least 54 subjects, according to a previous study [17], with a power of 80% and a confidence level of 95%.

## Results

**Fig. 1:**
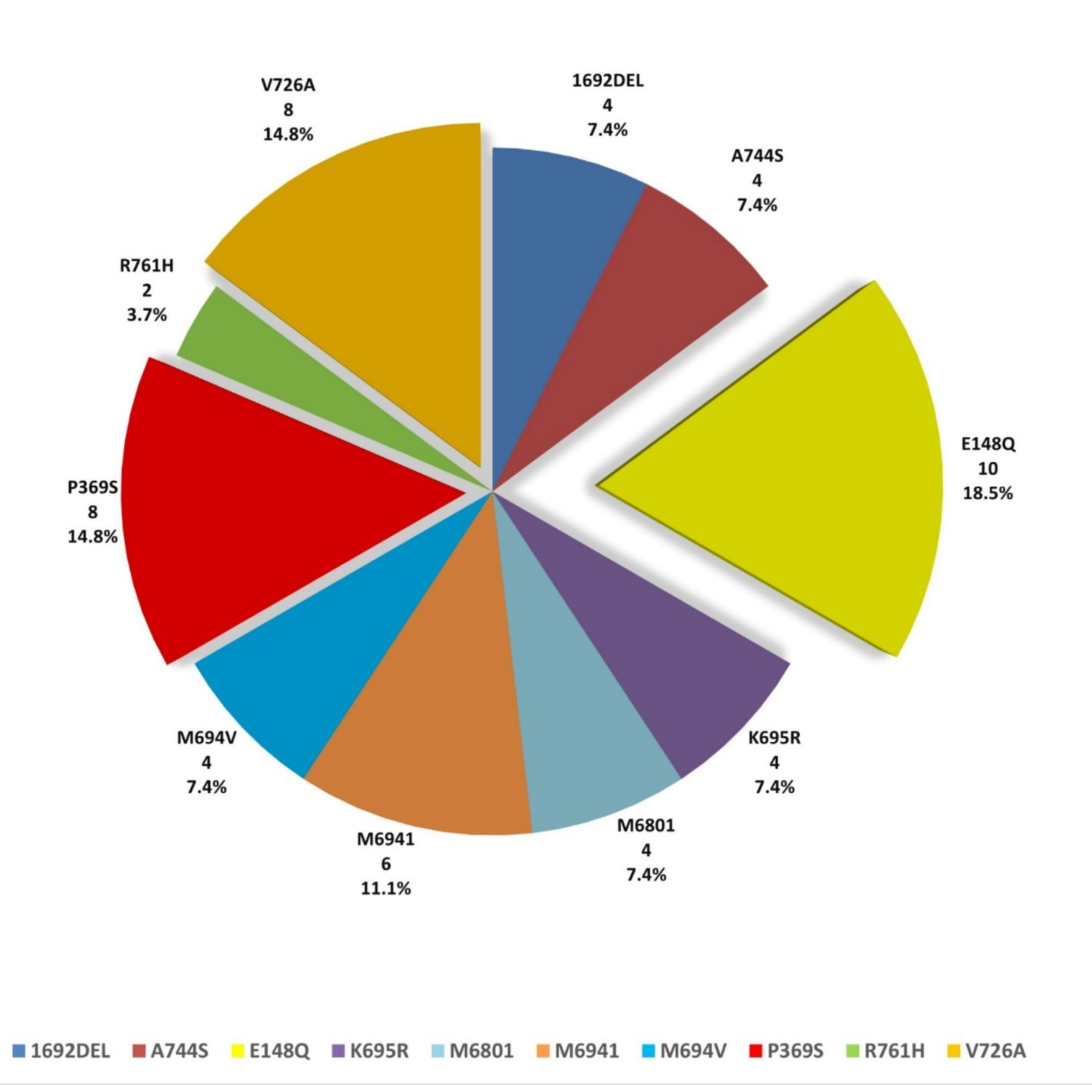
Distribution of MEFV gene mutations among the studied patients

Children with FMF had a mean age of 7.48 ± 2.96 years, which was comparable to that of the controls, 7.81 ± 3.11 years, with no statistical significance (*p* = 0.584). 28 patients were males (51.9%) and 26 were females (48.1%), compared to 30 (55.6%) and 24 (44.4%) in controls, respectively, with no statistical difference (*p* = 0.700). The body weight, height, and body mass index z-scores of the patients were statistically lower than those of the controls (-0.66 ± 0.44, -0.71 ± 0.53, and − 0.30 ± 0.69 versus 0.38 ± 0.35, 0.40 ± 0.40, and 0.32 ± 0.54, with *p* < 0.001, *p* < 0.001, *p* < 0.001, respectively). E148Q, P369S, and V726A were the most common MEFV gene mutations in patients, representing 18.5%, 14.8%, and 14.8%, respectively (Fig. 1).

Regarding laboratory data, patients during attack-free periods exhibited statistically higher total leucocytic counts (TLC), ESR, CRP, SAA, HOMA-IR, TC, non-HDL-C, CRI I, and AC, and a lower hemoglobin level than controls. During acute attacks, patients had a significantly higher TLC, hemoglobin level, platelet count, ESR, CRP, SAA, HOMA-IR, TC, LDL-C, TG, non-HDL-C, CRI I, CRI II, AIP, and AC, and lower HDL-C compared to attack-free periods (Table 1).

**Table 1:** Comparison between patients and controls regarding the studied laboratory parameters

	Patients during acute attacks (*n* = 54)	Patients during attack-free periods (n = 54)	Controls (n = 54)	*p* value
Total leucocytic count (10^3^/mm^3^) Mean ± SD	9.13 ± 3.04	8.79 ± 1.48	6.14 ± 0.99	p1 = 0.276 p2 < 0.001^*^ p3 = < 0.001
Hemoglobin(g/dl) Mean ± SD	11.68 ± 1.17	11.50 ± 1.04	11.98 ± 1.06	p1 = 0.001^*^ p2 < 0.001^*^ p3 = 0.037
Platelets (10^3^/mm^3^) Mean ± SD	293.2 ± 88.97	272.1 ± 48.99	282.5 ± 56.92	p1 = 0.001^*^ p2 = 0.971 p3 = 0.293
Erythrocyte sedimentation rate (mm/hr) Mean ± SD	27.44 ± 7.87	8.41 ± 2.21	7.22 ± 3.15	p1 < 0.001^*^ p2 < 0.001^*^ p3 = 0.020
C-reactive protein (mg/dl) Mean ± SD	55.09 ± 28.13	3.83 ± 1.73	2.75 ± 1.64	p_1_ < 0.001^*^ p_2_ < 0.001^*^ p_3_ = 0.002^*^
Serum amyloid A (mg/l) Mean ± SD	126.2 ± 42.17	6.46 ± 1.64	3.45 ± 1.51	p_1_ < 0.001^*^ p_2_ < 0.001^*^ p_3_ < 0.001^*^
HOMA-IR Mean ± SD	2.97 ± 0.64	1.26 ± 0.29	1.03 ± 0.23	p_1_ < 0.001^*^ p_2_ < 0.001^*^ p_3_ < 0.001^*^
Total cholesterol (mg/dl) Mean ± SD	199.1 ± 48.87	141.85 ± 25.43	115.1 ± 18.17	p_1_ < 0.001^*^ p_2_ < 0.001^*^ p_3_ = < 0.001^*^
Triglycerides (mg/dl) Mean ± SD	180.0 ± 75.13	109.07 ± 23.51	106.3 ± 45.61	p_1_ < 0.001^*^ p_2_ < 0.001^*^ p_3_ = 0.214
LDL (mg/dl) Mean ± SD	141.8 ± 23.23	97.74 ± 13.12	103.0 ± 18.65	p_1_ < 0.001^*^ p_2_ < 0.001^*^ p_3_ = 0.061
HDL (mg/dl) Mean ± SD	28.96 ± 4.24	45.67 ± 5.10	47.96 ± 11.33	p_1_ < 0.001^*^ p_2_ < 0.001^*^ p_3_ = 0.853
non-HDL (mg/dl) Mean ± SD	170.1 ± 49.95	96.19 ± 25.12	67.19 ± 24.38	p_1_ < 0.001^*^ p_2_ < 0.001^*^ p_3_ < 0.001^*^
CRI I Mean ± SD	7.06 ± 2.14	3.13 ± 0.61	2.54 ± 0.71	p_1_ < 0.001^*^ p_2_ < 0.001^*^ p_3_ < 0.001^*^
CRI II Mean ± SD	4.96 ± 0.92	2.16 ± 0.35	2.24 ± 0.61	p_1_ < 0.001^*^ p_2_ < 0.001^*^ p_3_ = 0.383
AIP Mean ± SD	0.76 ± 0.19	0.37 ± 0.11	0.32 ± 0.22	p_1_ < 0.001^*^ p_2_ < 0.001^*^ p_3_ = 0.127
AC Mean ± SD	6.06 ± 2.14	2.13 ± 0.61	1.54 ± 0.71	p_1_ < 0.001^*^ p_2_ < 0.001^*^ p_3_ < 0.001^*^

AIP; atherogenic index of plasma AC; atherogenic coefficient CRI I; Castelli’s risk index I CRI II; Castelli’s risk index II

HOMA-IR; Homeostatic Model Assessment for Insulin Resistance HDL; high density lipoprotein cholesterol LDL; low density lipoprotein cholesterol non-HDL; non-high density lipoprotein cholesterol SD: Standard deviation

p1: p value for comparing between patients during acute attacks and during attack-free periods

p2: p value for comparing patients during acute attacks and controls

p_3_: *p* value for comparing patients during attack-free periods and controls *: Statistically significant at *p* ≤ 0.05

Regarding NC changes, all controls exhibited no NC changes, which reflected significant detections in patients during attack-free periods, with *p* = 0.006, *p* < 0.001, *p* < 0.001, and *p* = 0.118 for avascular areas, tortuosities, enlargements, and microhemorrhages, respectively. In patients, avascular regions and tortuosities during acute attacks and attack-free periods did not show statistical significance. Nevertheless, capillary enlargements and microhemorrhages were significantly detected during acute attacks. From the lowest to the highest possible score, more patients with scores of 0 were recorded during attack-free periods, and the difference was statistically significant (Table 2).

**Table 2:** Comparison of nailfold capillaroscopic scoring in patients during acute attacks and attack-free periods

	Nailfold capillary changes during acute attacks		Nailfold capillary changes during attack-free periods		*p* value
	No.	%	No.	%	
Avascular area					
0	46	85.2	46	85.2	1.000
2	8	14.8	8	14.8	
Tortuosity					
0	40	74.1	44	81.5	0.125
1	10	18.5	10	18.5	1.000
2	4	7.4	0	0.0	0.125
Enlargements					
0	38	70.4	46	85.2	0.388
1	12	22.2	8	14.8	0.125
2	4	7.4	0	0.0	0.008^*^
Microhemorrhages					
0	42	77.8	50	92.6	0.008^*^
1	4	7.4	4	7.4	1.000
2	8	14.8	0	0.0	0.008^*^
Total scoring					
0	36	66.7	44	81.5	0.008^*^
1	4	7.4	0	0.0	0.125
2	2	3.7	0	0.0	0.500
3	2	3.7	4	7.4	0.687
4	0	0.0	4	7.4	0.125
5	4	7.4	2	3.7	0.687
6	4	7.4	0	0.0	0.125
7	0	0.0	0	0.0	–
8	2	3.7	0	0.0	0.500

*: Statistically significant at *p* ≤ 0.05

Patients with positive NC changes had a significantly longer disease duration, and a significantly higher SAA either during acute attacks or attack-free periods. Meanwhile, CRP was only significantly higher during acute attacks, and HDL-C was significantly higher during attack-free periods. Otherwise, Table 3 shows that no notable variations were observed in the other laboratory measures.

**Table 3:** Relations between patients’ nailfold capillary changes and the other studied variables during acute attacks and attack-free periods

	Nailfold capillary changes during acute attacks				Nailfold capillary changes during attack-free periods		
	negative (*n* = 36)	positive (*n* = 18)	*p* value	negative (*n* = 44)		positive (*n* = 10)	*p* value
Disease duration (years) Mean ± SD	2.06 ± 2.06	3.79 ± 2.10	0.010^*^	2.15 ± 1.99		4.80 ± 1.84	0.002^*^
C-reactive protein (mg/dl) Mean ± SD	48.14 ± 25.48	69.0 ± 28.70	0.004^*^	3.73 ± 1.69		4.30 ± 1.95	0.309
Serum amyloid A (mg/l) Mean ± SD	119.2 ± 36.79	140.2 ± 49.50	0.040^*^	6.24 ± 1.62		7.46 ± 1.34	0.040^*^
HOMA IR Mean ± SD	2.87 ± 0.60	3.16 ± 0.70	0.128	1.25 ± 0.30		1.30 ± 0.20	0.533
Total cholesterol (mg/dl) Mean ± SD	193.2 ± 41.61	210.8 ± 60.53	0.557	142.8 ± 25.70		137.8 ± 25.10	0.655
Triglycerides (mg/dl) Mean ± SD	176.1 ± 77.73	187.8 ± 71.13	0.398	108.1 ± 24.66		113.6 ± 17.96	0.448
LDL (mg/dl) Mean ± SD	142.4 ± 20.43	140.7 ± 28.65	0.741	96.73 ± 14.05		102.2 ± 6.55	0.283
HDL (mg/dl) Mean ± SD	29.22 ± 4.92	28.44 ± 2.43	0.971	44.82 ± 4.48		49.40 ± 6.17	0.022^*^
non-HDL Mean ± SD	164.0 ± 43.33	182.3 ± 60.64	0.207	97.95 ± 24.71		88.40 ± 26.75	0.282
CRI I Mean ± SD	6.85 ± 2.02	7.47 ± 2.36	0.323	3.20 ± 0.57		2.84 ± 0.72	0.094
CRI II Mean ± SD	4.96 ± 0.87	4.97 ± 1.04	0.984	2.17 ± 0.35		2.10 ± 0.33	0.563
AIP Mean ± SD	0.75 ± 0.19	0.79 ± 0.19	0.426	0.37 ± 0.12		0.36 ± 0.09	0.752
AC Mean ± SD	5.85 ± 2.02	6.47 ± 2.36	0.323	2.20 ± 0.57		1.84 ± 0.72	0.094

AIP; atherogenic index of plasma AC; atherogenic coefficient CRI I; Castelli’s risk index I CRI II; Castelli’s risk index II

HOMA-IR; Homeostatic Model Assessment for Insulin Resistance HDL; high density lipoprotein cholesterol LDL; low density lipoprotein cholesterol non-HDL; non-high density lipoprotein cholesterol SD: Standard deviation

*: Statistically significant at *p* ≤ 0.05

**Fig. 2:**
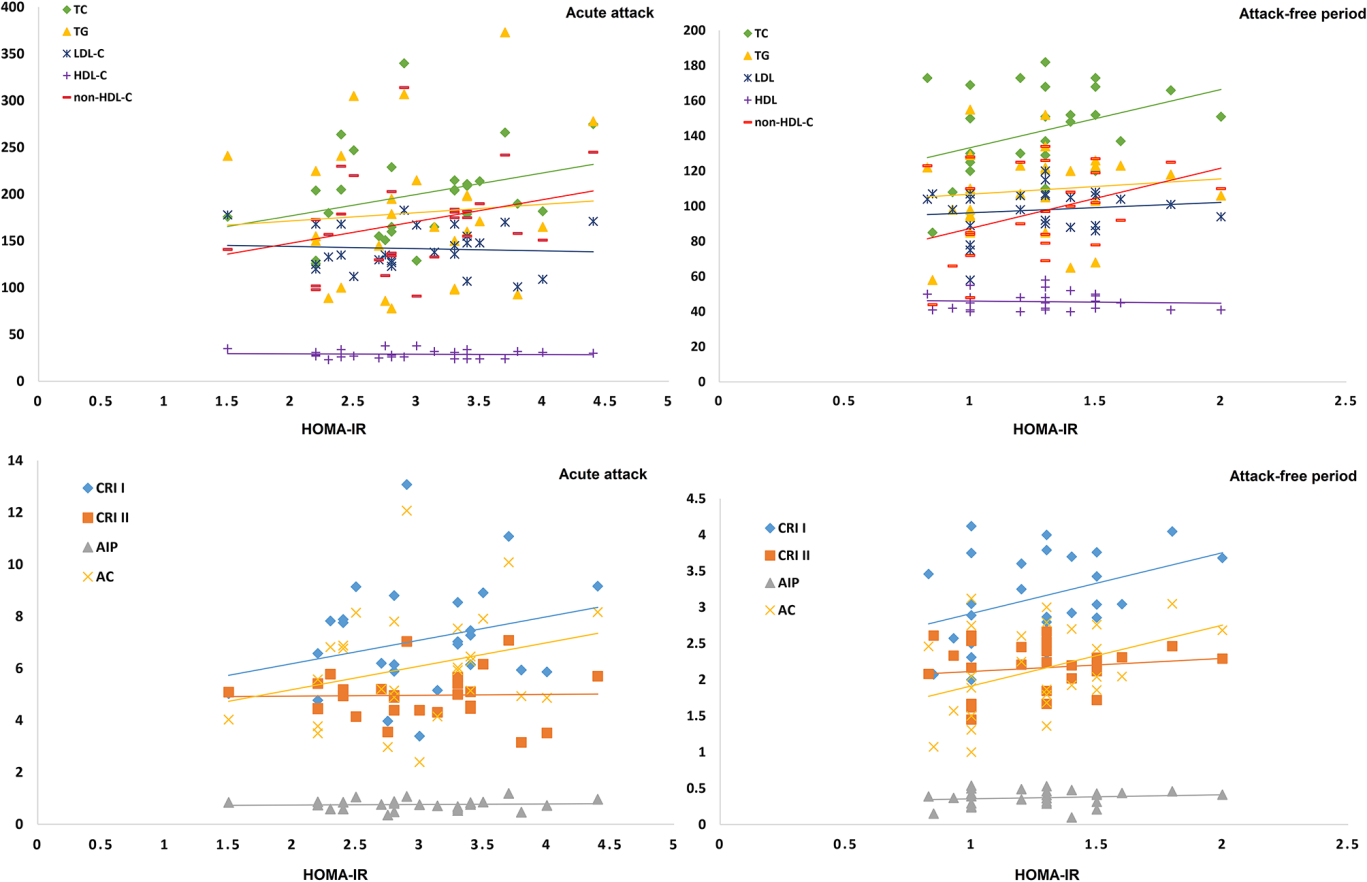
Correlations between HOMA-IR, serum lipid parameters, and atherogenic indices in patients during acute attacks and attack-free periods of the disease. (AIP; atherogenic index of plasma AC; atherogenic coefficient CRI I; Castelli’s risk index I CRI II; Castelli’s risk index II HOMA-IR; Homeostatic Model Assessment for Insulin Resistance HDL; high density lipoprotein cholesterol LDL; low density lipoprotein cholesterol non-HDL; non-high density lipoprotein cholesterol TC; Total cholesterol TG; Triglycerides)

Regarding correlations between the parameters studied, positive NC changes were substantially linked with disease duration, platelets, TLC, and SAA either during acute attacks or attack-free periods, with CRP and HOMA-IR during acute attacks, and with HDL-C, CRI I, and AC during attack-free periods. Moreover, HOMA-IR correlated positively with TC, non-HDL-C, CRI I, and AC both during acute attacks and attack-free periods, and with TLC, platelet count, CRP, and SAA only during acute attacks. Additionally, CRP correlated positively with TC, non-HDL-C, CRI I, CRI II, and AC, and negatively with HDL-C levels during acute attacks (Table 4; Fig. 2).

**Table 4:** Correlations between nailfold capillary changes, HOMA-IR, C-reactive protein, and serum amyloid A and different parameters in patients during acute attacks and attack-free periods

	Nailfold capillary changes				HOMA-IR				C-reactive protein				Serum amyloid A			
	Acute attacks		Attack-free periods		Acute attacks		Attack-free periods		Acute attacks		Attack-free periods		Acute attacks		Attack-free periods	
	r	p	r	p	r	p	r	p	r_s_	p	r	p	r_s_	p	r_s_	p
Disease duration (years)	**0.437**	**0.001** ^*^	**0.485**	**< 0.001** ^*^	0.174	0.207	0.087	0.530	-0.130	0.349	-0.061	0.661	0.272	0.047	0.155	0.264
Total cholesterol	0.126	0.366	-0.158	0.254	**0.302**	**0.026** ^*^	**0.373**	**0.005** ^*^	**0.465**	**< 0.001** ^*^	0.080	0.565	0.056	0.689	-0.161	0.244
Triglycerides	0.054	0.697	0.062	0.658	0.076	0.583	0.106	0.444	0.114	0.411	0.045	0.745	-0.049	0.724	0.002	0.989
LDL	-0.206	0.135	0.179	0.195	-0.065	0.639	0.130	0.349	0.156	0.260	-0.162	0.241	-0.191	0.167	0.012	0.930
HDL	-0.107	0.442	**0.351**	**0.009** ^*^	-0.059	0.672	-0.068	0.628	**-0.405**	**0.002** ^*^	0.103	0.461	-0.213	0.122	0.200	0.148
non-HDL	0.132	0.342	-0.231	0.093	**0.301**	**0.027** ^*^	**0.392**	**0.003** ^*^	**0.501**	**< 0.001** ^*^	0.032	0.821	0.083	0.550	-0.204	0.139
CRI I	0.118	0.394	**-0.305**	**0.025** ^*^	**0.272**	**0.047** ^*^	**0.395**	**0.003** ^*^	**0.576**	**< 0.001** ^*^	0.002	0.990	0.179	0.195	-0.250	0.068
CRI II	-0.126	0.365	-0.073	0.600	0.023	0.868	0.150	0.280	**0.380**	**0.005** ^*^	**-0.327**	**0.016** ^*^	-0.079	0.568	0.032	0.818
AIP	0.109	0.432	-0.074	0.593	0.071	0.608	0.146	0.293	0.210	0.127	-0.051	0.715	-0.003	0.984	-0.054	0.696
AC	0.118	0.394	**-0.305**	**0.025** ^*^	**0.272**	**0.047** ^*^	**0.395**	**0.003** ^*^	**0.576**	**< 0.001** ^*^	0.002	0.990	0.179	0.195	-0.250	0.068
Total leucocytic count	**0.709**	**< 0.001** ^*^	**0.699**	**< 0.001** ^*^	**0.308**	**0.023** ^*^	0.132	0.340	0.252	0.066	0.049	0.723	**0.368**	**0.006** ^*^	0.182	0.188
Hemoglobin	0.164	0.237	0.200	0.148	0.007	0.963	-0.199	0.150	-0.111	0.424	0.204	0.140	-0.001	0.995	0.144	0.298
Platelets	**0.436**	**0.001** ^*^	**0.582**	**< 0.001** ^*^	**0.334**	**0.014** ^*^	-0.193	0.163	**0.428**	**0.001** ^*^	0.130	0.350	0.138	0.321	-0.132	0.342
ESR	0.235	0.088	0.250	0.068	0.349	0.010^*^	-0.265	0.053	**0.327**	**0.016** ^*^	0.134	0.336	0.175	0.205	0.149	0.284
Serum amyloid A	**0.349**	**0.010** ^*^	**0.312**	0.022*	**0.626**	**< 0.001** ^*^	-0.110	0.429	**0.579**	**< 0.001** ^*^	0.004	0.975	-------	-------	-------	-------
C-reactive protein	**0.430**	**0.001** ^*^	0.150	0.278	**0.402**	**0.003** ^*^	0.110	0.428	-------	-------	-------	-------	**0.579**	**< 0.001** ^*^	0.004	0.975
HOMA-IR	**0.360**	**0.007** ^*^	0.040	0.772	-------	-------	-------	-------	**0.656**	**< 0.001** ^*^	-0.113	0.417	**0.626**	**< 0.001** ^*^	-0.110	0.429
Nailfold capillary changes	-------	-------	-------	-------	**0.360**	**0.007** ^*^	0.040	0.772	**0.430**	**0.001** ^*^	0.150	0.278	**0.349**	**0.010** ^*^	**0.312**	**0.022** ^*^

AIP; atherogenic index of plasma AC; atherogenic coefficient CRI I; Castelli’s risk index I CRI II; Castelli’s risk index II ESR; Erythrocyte sedimentation rate HOMA-IR; Homeostatic Model Assessment for Insulin Resistance HDL; high density lipoprotein cholesterol LDL; low density lipoprotein cholesterol non-HDL; non-high density lipoprotein cholesterol p: *p* value *: Statistically significant at p ≤ 0.05 r = Pearson coefficient, r_s_: Spearman coefficient

In univariate regression analysis, disease duration, TLC, platelet count, SAA, HDL-C, and AC were substantially related to the prediction of NC changes during attack-free periods, and only TLC, platelet count, and SAA retained a significant reliance in the prediction by multivariate analysis. Regarding acute attacks, disease duration, TLC, and CRP retained significant predictive value for NC changes, surpassing SAA and HOMA-IR in multivariate analysis (Table 5).

**Table 5:** Univariate and multivariate linear regression analyses for the prediction of the variables that may affect nailfold capillary changes in patients during acute attacks and attack-free periods

	Univariate		Multivariate	
	p	B (LL – UL 95% C.I)	p	B (LL – UL 95% C.I)
During attack-free periods				
Disease duration (years)	< 0.001*	0.335(0.167–0.503)	**0.989**	-0.004(-0.535–0.528)
Total leucocytic count	< 0.001*	0.350(0.251–0.450)	0.002*	0.190(0.076–0.305)
Platelets	< 0.001*	0.010(0.006–0.014)	0.007*	0.005(0.001–0.009)
Serum amyloid A	0.014*	0.310(0.066–0.555)	0.010*	0.224(0.056–0.392)
HDL	0.009*	0.105(0.027–0.183)	**0.125**	0.046(-0.013–0.106)
AC	0.025*	-0.763(-1.427 – -0.099)	**0.697**	-0.100(-0.617–0.416)
During acute attacks				
Disease duration (years)	0.001*	0.471(0.201–0.740)	0.037*	0.937(0.057–1.816)
Total leucocytic count	< 0.001*	0.555(0.402–0.708)	0.001*	0.356(0.165–0.547)
Platelets	0.001*	0.012(0.005–0.018)	**0.486**	-0.002(-0.009–0.004)
C-reactive protein	0.001*	0.037(0.016–0.058)	0.004*	0.046(0.016–0.076)
Serum amyloid A	0.004*	0.022(0.008–0.037)	**0.192**	-0.010(-0.025–0.005)
HOMA-IR	0.007*	1.333(0.373–2.292)	**0.898**	0.059(-0.855–0.973)

AC; atherogenic coefficient B; Unstandardized Coefficients C.I; Confidence interval HOMA-IR; Homeostatic Model Assessment for Insulin Resistance HDL; high density lipoprotein cholesterol LL; Lower limit UL; Upper Limit p: *p* value *: Statistically significant at *p* ≤ 0.05

## Discussion

The present study demonstrated that insulin resistance, alterations in serum lipids and atherogenic indices, and NC changes develop during FMF attack-free periods, and significantly increase during acute attacks. These findings may indicate that FMF patients are at higher risk for cardiovascular disease, especially with early life presentation.

Atherosclerosis is a major contributor to worldwide morbidity and mortality. It begins early in life, advances gradually and asymptomatically with age, and finally causes unfavorable cardiovascular events. While age, insulin resistance, hypertension, dyslipidemia, and smoking are classic risk factors, genetics, environmental variables, and autoinflammatory disorders also play a part in this process [18–20].

FMF is a chronic inflammatory disease caused by mutations in the MEFV gene, which encodes the pyrin protein that is thought to play a role in the inflammatory pathways. These mutations can lead to a loss of pyrin function, resulting in uncontrolled inflammation, which may contribute to endothelial dysfunction, prothrombotic conditions, and functional changes in microcirculation. These changes could impact the extensibility and flexibility of the arterial wall, ultimately resulting in atherosclerotic consequences [21–23].

FMF and insulin resistance are mutually correlated with chronic subclinical inflammation. There is even a possibility that long-term inflammation could trigger the development of insulin resistance [7, 24]. It is commonly known that insulin resistance promotes endothelial dysfunction and inflammation directly by preventing the synthesis of nitric oxide, which, in turn, causes atherosclerotic cardiovascular disease [25].

Numerous studies have reported a correlation between arterial stiffness and insulin resistance, as measured by the HOMA-IR in various demographic groups, in which asymptomatic individuals may experience remarkable vascular damage that is not supported by traditional cardiovascular risk factors like smoking or hypercholesterolemia. Even HOMA-IR values have been independently linked to higher arterial stiffness in healthy children and adolescents, as measured by brachial artery distensibility or carotid-femoral pulse-wave velocity, highlighting an intimate link between insulin resistance and early-onset vascular damage [6, 26–28].

Studies pertaining to insulin resistance and FMF are scarce. A pediatric study by Atef et al. [29] and an adult study by Sarkis et al. [7] reported that patients with FMF exhibited increased insulin resistance with significantly higher HOMA-IR compared to the controls. Nevertheless, they did not determine whether this increase occurred during acute attacks, attack-free periods, or both. Conversely, Ugurlu et al. [30] were unable to find any differences in insulin resistance between adult FMF patients and controls. Additionally, Dursun et al. [17] did not observe evident insulin resistance in children with FMF.

Regarding our study, patients’ HOMA-IR values increased significantly during attack-free periods relative to the controls, and they increased even more during acute attacks. This strengthens the idea that FMF inflammatory pathways and insulin resistance are tightly connected, and it suggests the necessity for more extensive longitudinal research.

A higher risk of accelerated atherosclerosis has been linked to changes in serum lipids in inflammatory disorders such as rheumatoid arthritis, systemic lupus erythematosus, and ankylosing spondylitis. High TG levels and low TC, LDL-C, and HDL-C levels were among the characteristic changes observed. Still, controlling disease activity helps to improve dyslipidemia [31–33].

Studies on FMF patients have shown a statistically significant reduction in HDL-C levels both during the acute attacks and attack-free periods. Others have shown that low HDL-C levels were present in even first-degree FMF relatives who did not exhibit symptoms. It was also noted that FMF patients had greater TG levels [31, 34, 35]. El Zayat et al. [4] highlighted that LDL-C and TG were significantly higher in patients, particularly in those with homozygous mutations.

In this instance, our patients exhibited statistically significant elevations in TC and non-HDL-C levels during attack-free periods relative to the controls, as well as statistically significant elevations in TC, LDL-C, and non-HDL-C levels, with significantly lower HDL-C levels during acute attacks. These findings resemble a traditional atherogenic lipid profile, which underscores the necessity of keeping a constant eye on serum lipid concentrations to identify any abnormalities early and take necessary corrective measures.

Recent research has identified atherogenic indices as important indicators of atherosclerosis, as they are less susceptible to changes in disease activity over extended periods of time. This makes them a more appealing option in cardiovascular risk prediction in this patient population [36–38]. In angiographically verified cases of coronary artery disease (CAD), Bhardwaj et al. [39] found that AIP, CRI I, CRI II, and AC were highly elevated and markedly increased the CAD risk by up to 31%, 20%, 20%, and 17%, respectively.

In adults, Çakırca et al. [8] demonstrated that CRI I and II, AIP, and AC values were significantly higher in FMF patients than in controls. Furthermore, greater AIP values in FMF patients were observed by Icli et al. [40], with a positive connection to carotid intima-media thickness, a marker of preclinical atherosclerosis. In children, El Zayat et al. [4] reported statistically higher CRI II and AIP in FMF patients than in controls. Vampertzi et al. [41] observed significant elevations in TG levels and AIP values in children and young adults with FMF compared to controls. Nevertheless, none of the previous studies examined these parameters during both acute attacks and attack-free periods.

In our study, patients’ CRI I and AC during attack-free periods were significantly higher than those of the controls, and their CRI I, CRI II, AIP, and AC during acute attacks were significantly greater than those during attack-free periods. Given that these markers are less sensitive to changes in disease activity, easily obtained through ordinary biochemical measures, and are rising valuable markers in the prediction of cardiovascular disease, larger longitudinal studies are needed to elucidate the significance of atherogenic indices in FMF patients, especially in youngsters, given the substantial impact early-onset atherosclerosis would have on adult lives.

Nailfold capillaroscopy has recently grown in the study of peripheral microangiopathy, offering significant preclinical insights into vascular health, particularly in the field of rheumatology [42, 43]. In adults, it aids in the diagnosis of connective tissue diseases and in distinguishing between primary and secondary Raynaud’s phenomenon [44–46]. The European Alliance of Associations for Rheumatology on microcirculation in rheumatic diseases recommended international collaboration for nailfold capillaroscopic data collection from children and adolescents with and without juvenile rheumatic and musculoskeletal diseases [42, 43]. Despite this, it is less commonly used in children and adolescents, and the related studies are scarce.

Aytekin et al. [47] reported NC abnormalities with focal enlargements in 16.13% and microhemorrhages in 3.23% of adult patients with FMF who had no evidence of vasculitis during their study. Dinc et al. [16] found tortuosities in 28.8%, microhemorrhages in 23.7%, and enlargements in 15.3% of adult FMF patients; however, these changes were more commonly seen in patients accompanied by Raynaud’s phenomenon. In children, Dursun et al. [17] reported NC changes in 20% of patients during acute attacks and in 2.9% of patients during attack-free periods; however, the patients studied were not the same during the acute attacks and attack-free periods of the disease.

Herein, our patients documented NC changes both during acute attacks and attack-free periods of the disease, with significant detection during acute attacks, in which avascular areas were detected in 14.8% of patients, tortuosities in 25.9%, enlargements in 29.6%, and microhemorrhages in 22.2% compared to 14.8%, 18.5%, 14.8%, and 7.4% during attack-free periods, respectively. Consequently, our findings imply that the NC changes may persist throughout the FMF attack-free periods, pointing to the potential for peripheral microangiopathy.

Beyond that, research has not sufficiently examined how these variables relate to one another in FMF patients. In our study, HOMA-IR values showed significant elevations in patients during acute attacks and attack-free periods, with strong correlations to TC, non-HDL-C, CRI I, and AC. NC changes endured in patients during attack-free periods, with significant increase during acute attacks, especially with longer disease duration, and were strongly associated with significant elevations in TLC, platelet count, CRP, SAA, and HOMA-IR during acute attacks, suggesting possibility of underlying peripheral microangiopathy.

This concludes that the development of insulin resistance, abnormal serum lipids, and NC changes in children with FMF might help in the early detection of patients with microangiopathy who are more susceptible to cardiovascular complications later in life. If such high-risk patients are identified early, this could help the physician to provide a closer follow-up and adjust the treatment strategy as needed. Consequently, we advise conducting additional extensive research on these variables in children with FMF during both acute attacks and attack-free periods and connecting them to a cardiovascular clinical maneuver or imaging method for more validation.

Regarding limitations encountered in our study, the sample size and the lack of studies in this field for comparison were the main restrictions faced. Moreover, many children were excluded either due to associated hand trauma or because the diagnosis of Raynaud’s phenomenon could not be ruled out. Additionally, all patients were on colchicine therapy, which has a contentious effect on the serum lipid profile. Nevertheless, to the best of our knowledge, we are the first to examine insulin resistance, lipid profiles, atherogenic indices, and NC changes in children with FMF, both during acute attacks and attack-free periods in the same patients.

## Conclusion

Children with FMF are susceptible to increased insulin resistance both during active and non-active periods of the disease, which may be accompanied by alterations in serum lipid profiles and substantial elevations in atherogenic indices. These parameters are intimately linked to atherosclerotic changes and increased cardiovascular risk later in life, which recommends close follow-up of these patients for early intervention and prevention of any future complications. Additionally, NC changes occur in children with FMF even during non-active periods of the disease, and may reflect underlying peripheral microangiopathy, which can be easily detected through nailfold capillaroscopic examination. Therefore, further large-scale studies in various demographics will help in identifying patterns of NC changes in these patients, and their relations to FMF inflammatory process and underlying genetic patterns.

## Data Availability

The datasets used and/or analyzed during the current study are available from the corresponding author on reasonable request.

## Abbreviations

AC: Atherogenic coefficient
AIP: Atherogenic index of plasma
CAD: Coronary artery disease
CRI I: Castelli’s risk index I
CRI II: Castelli’s risk index II
CRP: C reactive protein
FMF: Familial Mediterranean fever
HDL-C: High-density lipoprotein cholesterol
HOMA-IR: Homeostatic Model Assessment for Insulin Resistance
LDL-C: Low density lipoprotein cholesterol
MEFV: Mediterranean fever gene
NC: Nailfold capillary
non-HDL-C: Non-high-density lipoprotein cholesterol
SAA: Serum amyloid A
TC: Total cholesterol
TG: Triglycerides
TLC: Total leucocytic count
